# Association Analysis of the *FTO* Gene with Obesity in Children of Caucasian and African Ancestry Reveals a Common Tagging SNP

**DOI:** 10.1371/journal.pone.0001746

**Published:** 2008-03-12

**Authors:** Struan F. A. Grant, Mingyao Li, Jonathan P. Bradfield, Cecilia E. Kim, Kiran Annaiah, Erin Santa, Joseph T. Glessner, Tracy Casalunovo, Edward C. Frackelton, F. George Otieno, Julie L. Shaner, Ryan M. Smith, Marcin Imielinski, Andrew W. Eckert, Rosetta M. Chiavacci, Robert I. Berkowitz, Hakon Hakonarson

**Affiliations:** 1 Center for Applied Genomics, Abramson Research Center, The Children's Hospital of Philadelphia, Philadelphia, Pennsylvania, United States of America; 2 Division of Human Genetics, Department of Pediatrics, The Children's Hospital of Philadelphia, Philadelphia, Pennsylvania, United States of America; 3 Department of Pediatrics, University of Pennsylvania School of Medicine, Philadelphia, Pennsylvania, United States of America; 4 Department of Biostatistics and Epidemiology, University of Pennsylvania, Philadelphia, Pennsylvania, United States of America; 5 Weight and Eating Disorders Program, Department of Psychiatry, University of Pennsylvania School of Medicine, Philadelphia, Pennsylvania, United States of America; University of Bremen, Germany

## Abstract

Recently an association was demonstrated between the single nucleotide polymorphism (SNP), rs9939609, within the *FTO* locus and obesity as a consequence of a genome wide association (GWA) study of type 2 diabetes in adults. We examined the effects of two perfect surrogates for this SNP plus 11 other SNPs at this locus with respect to our childhood obesity cohort, consisting of both Caucasians and African Americans (AA). Utilizing data from our ongoing GWA study in our cohort of 418 Caucasian obese children (BMI≥95th percentile), 2,270 Caucasian controls (BMI<95th percentile), 578 AA obese children and 1,424 AA controls, we investigated the association of the previously reported variation at the *FTO* locus with the childhood form of this disease in both ethnicities. The minor allele frequencies (MAF) of rs8050136 and rs3751812 (perfect surrogates for rs9939609 i.e. both r^2^ = 1) in the Caucasian cases were 0.448 and 0.443 respectively while they were 0.391 and 0.386 in Caucasian controls respectively, yielding for both an odds ratio (OR) of 1.27 (95% CI 1.08–1.47; *P* = 0.0022). Furthermore, the MAFs of rs8050136 and rs3751812 in the AA cases were 0.449 and 0.115 respectively while they were 0.436 and 0.090 in AA controls respectively, yielding an OR of 1.05 (95% CI 0.91–1.21; *P* = 0.49) and of 1.31 (95% CI 1.050–1.643; *P* = 0.017) respectively. Investigating all 13 SNPs present on the Illumina HumanHap550 BeadChip in this region of linkage disequilibrium, rs3751812 was the only SNP conferring significant risk in AA. We have therefore replicated and refined the association in an AA cohort and distilled a tag-SNP, rs3751812, which captures the ancestral origin of the actual mutation. As such, variants in the *FTO* gene confer a similar magnitude of risk of obesity to children as to their adult counterparts and appear to have a global impact.

## Introduction

Obesity is a major health problem in modern societies. Approximately 127 million adults in the U.S. are overweight (BMI>25), 60 million are obese (BMI>30), and 9 million are severely obese (BMI>40). Obesity also shows increasing prevalence in children. Childhood obesity is considered to have reached epidemic levels in developed countries. In the 10 years between the National Health and Nutrition Examination Survey (NHANES) II (1976–1980) and NHANES III (1988–1991), the prevalence of overweight children in the USA had increased by approximately 40%[1].

Approximately 70% of obese adolescents grow up to become obese adults[2], [3], [4]. The main direct adverse effects of childhood obesity include orthopedic complications, sleep apnea, and psychosocial disorders[5], [6]. Obesity and its associated phenotype, insulin resistance[7], [8], is also considered a contributor to the major causes of death in the United States and is an important risk factor for type 2 diabetes (T2D), cardiovascular disease, hypertension and other chronic diseases[9]. Obesity present in adolescence has been shown to be associated with increased overall mortality in adults[10].

The Center for Disease Control and Prevention has defined overweight as BMI≥95^th^ percentile for each age group whereas European researchers use that same definition to classify obesity[11]. The Institute of Medicine also specifically uses the term “obesity” to characterize BMI≥95^th^ percentile in children and adolescents[12], and these statistical percentile definitions are now general guidelines for clinicians and others[13].

Whereas the rising prevalence of obesity can be partly explained by environmental changes over the last 30 years, in particular the unlimited supply of convenient, highly calorific foods together with a sedentary lifestyle, there is also strong evidence for a genetic component to the risk of obesity[14], [15]. The familial occurrences of obesity have long been noted. For example, the concordance for fat mass among monozygotic twins is reported to be 70–90%, higher than the 35–45% concordance in dizygotic twins[16], [17], suggesting the heritability of BMI ranges from 30 to 70%[18], [19], [20], [21]. The genetic impact on obesity is also reflected in prevalence differences between racial groups, from 5% or less in Caucasian and Asian populations to 50% or more among Pima Indians[22] and South Sea Island populations[23].

Recently, a number of studies have reported remarkably strong, replicable associations with complex disease, including the complement factor H (*CFH*) gene in age-related macular degeneration[24], [25], [26] and the transcription factor 7-like-2 (*TCF7L2*) gene in type 2 diabetes[27], [28], [29].

Although two genes for obesity have already been reported from the outcome of genome wide association (GWA) studies, the role of *INSIG2* gene[30] has proven to be controversial due to three subsequent technical comments to *Science* [31], [32], [33] that disagreed with this observation, while the identification of the second gene, *FTO* [NM_001080432][34], was made indirectly as a consequence of T2D GWA studies[35], [36]. This gene variant turned out to be operating through insulin resistance and was found to be associated with adult BMI, and subsequently pediatric BMI, in the original study. In an independent study of the genetics of BMI that came out around the same time, similar conclusions were drawn[37].

Although these *FTO* findings are compelling, there are continuing concerns regarding the performance of association studies in complex traits. As such, independent replication efforts are now considered mandatory[38]. With the many errors and biases that can blight any individual study, replication by others can ensure that the original findings are robust and can also provide a more accurate estimate of the likely effect size[39], [40].

In this study we demonstrate that two SNPs, rs8050136 and rs3751812, in the *FTO* gene confer a similar magnitude of risk for obesity in our pediatric cohort as previously reported in both adults and children with the same phenotype, further supporting the notion that the FTO pathway is causally linked to the disorder in children. In addition, we have replicated and refined the association in an African American (AA) cohort and distilled a tag-SNP, namely rs3751812, to capture the ancestral origin of the underlying mutational event.

## Results

### Association between rs8050136 and rs3751812 and childhood obesity risk in Caucasians

Using the allelic chi-squared association test, we observed significant association between rs8050136 and rs3751812 and risk for childhood obesity. The minor allele frequencies (MAFs) of these two SNPs in the Caucasian cases were 0.448 and 0.443, respectively, whereas the MAFs were 0.391 and 0.386, respectively, in the Caucasian controls, yielding for both an odds ratio (OR) of 1.27 (95% CI 1.08–1.47; *P* = 0.0022) (Table 1a). This OR is very much in line with that reported previously in the adult case-control cohort (OR = 1.2)[34].

**Table 1:** Childhood obesity case-control association study results for *FTO* region markers perfectly correlated with rs9939609 in Caucasians (bold) plus all other markers present on the BeadChip in the corresponding HapMap CEU region of LD in our (a) Caucasian and (b) African American cohorts respectively.

(a) Caucasians										
Chr	SNP	r^2^ to rs9939609	B35 location	Minor Allele	Major Allele	Aff MAF (n = 418)	Ctrl MAF (n = 2270)	OR	95% CI	*P*-value
16	rs9930333	0.843	52357478	T	G	0.476	0.425	1.228	1.058–1.427	0.0070
16	rs10852521	0.584	52362466	T	C	0.422	0.484	0.779	0.670–0.906	0.0011
16	rs16945088	0.099	52370025	G	A	0.094	0.090	1.056	0.818–1.365	0.68
**16**	**rs8050136**	**1**	**52373776**	**A**	**C**	**0.448**	**0.391**	**1.266**	**1.088–1.471**	**0.0022**
**16**	**rs3751812**	**1**	**52375961**	**T**	**G**	**0.443**	**0.386**	**1.267**	**1.089–1.473**	**0.0022**
16	rs12597786	0.014	52378808	T	C	0.026	0.020	1.320	0.815–2.138	0.26
16	rs9931164	0.022	52382739	G	A	0.020	0.019	1.024	0.597–1.756	0.93
16	rs9941349	0.9	52382989	T	C	0.462	0.407	1.254	1.080–1.457	0.0030
16	rs7199182	-	52383621	G	A	-	-	-	-	-
16	rs7190492	0.328	52386253	A	G	0.296	0.354	0.767	0.651–0.903	0.0014
16	rs8044769	0.62	52396636	T	C	0.414	0.483	0.758	0.652–0.881	0.00031
16	rs6499646	0.101	52401034	C	T	0.103	0.092	1.141	0.890–1.462	0.30
16	rs1421090	0.026	52407671	C	T	0.273	0.260	1.073	0.907–1.270	0.41

(b) African Americans										
Chr	SNP	r^2^ to rs9939609	B35 location	Minor Allele	Major Allele	Aff MAF (n = 578)	Ctrl MAF (n = 1424)	OR	95% CI	*P*-value
16	rs9930333	0.388	52357478	G	T	0.474	0.457	1.070	0.932–1.229	0.33
16	rs10852521	0.021	52362466	T	C	0.251	0.267	0.922	0.787–1.079	0.31
16	rs16945088	0.619	52370025	G	A	0.310	0.299	1.054	0.907–1.223	0.49
**16**	**rs8050136**	**0.819**	**52373776**	**A**	**C**	**0.449**	**0.437**	**1.051**	**0.914–1.207**	**0.49**
**16**	**rs3751812**	**0.058**	**52375961**	**T**	**G**	**0.115**	**0.090**	**1.313**	**1.050–1.643**	**0.017**
16	rs12597786	0.018	52378808	T	C	0.022	0.024	0.902	0.567–1.434	0.66
16	rs9931164	0.058	52382739	G	A	0.045	0.049	0.902	0.649–1.253	0.54
16	rs9941349	0.02	52382989	T	C	0.187	0.170	1.125	0.941–1.345	0.20
16	rs7199182	0.486	52383621	G	A	0.296	0.308	0.945	0.813–1.099	0.46
16	rs7190492	0.044	52386253	A	G	0.204	0.198	1.037	0.872–1.233	0.68
16	rs8044769	0.039	52396636	T	C	0.233	0.234	0.995	0.846–1.171	0.95
16	rs6499646	0.004	52401034	T	C	0.432	0.399	1.148	0.998–1.320	0.053
16	rs1421090	0.041	52407671	C	T	0.294	0.291	1.014	0.872–1.180	0.85

Minor allele frequencies (MAF), allelic test *P*-values, and odds ratios (OR) with 95% confidence intervals (CI) are shown for each SNP. rs7199182 has a MAF<1% in Caucasians so was not analyzed in that ethnicity. The ORs shown are for the minor allele. *P*-values are two-sided in each instance. r^2^ values to rs9939609 derived from (a) CEU and (b) YRI HapMap samples are also shown.

We also analyzed 11 additional SNPs on the Illumina 550K BeadChip in the region of LD harboring the association signal. Table 1a shows that five other SNPs that are in strong, but not in perfect LD with rs9939609 were also nominally associated with childhood obesity. Further analysis on haplotypes suggests that the major alleles of these SNPs are more likely to be on the same haplotype as the minor alleles of rs8050136 and rs3751812 (Table S1). The corresponding analysis of the entire cohort as a quantitative trait with these SNPs is shown in Table S2.

### Association between rs8050136 and rs3751812 and childhood obesity risk in African Americans

We also analyzed rs8050136 and rs3751812 in our 578 AA obese children and 1,424 AA controls (BMI<95th percentile). The MAFs in the AA cases were 0.449 and 0.115 respectively, whereas the MAFs were 0.436 and 0.090, respectively, in the AA controls, yielding an OR of 1.05 (95% CI 0.91–1.21; *P* = 0.49) and of 1.31 (95% CI 1.050–1.643; *P* = 0.017) (Table 1b). It is worth noting that rs8050136 shows strong LD with rs9939609 in the YRI sample (r^2^ = 0.819), whereas rs3751812 is only in weak LD (r^2^ = 0.058).

In addition, we analyzed the other 11 SNPs on the Illumina 550K BeadChip in the region of LD harboring the signal as seen in Caucasian samples. None of these additional SNPs showed nominally significant association to the phenotype (Table 1b). The corresponding analysis of the entire cohort as a quantitative trait with these SNPs is shown in Table S2.

**Figure 1:**
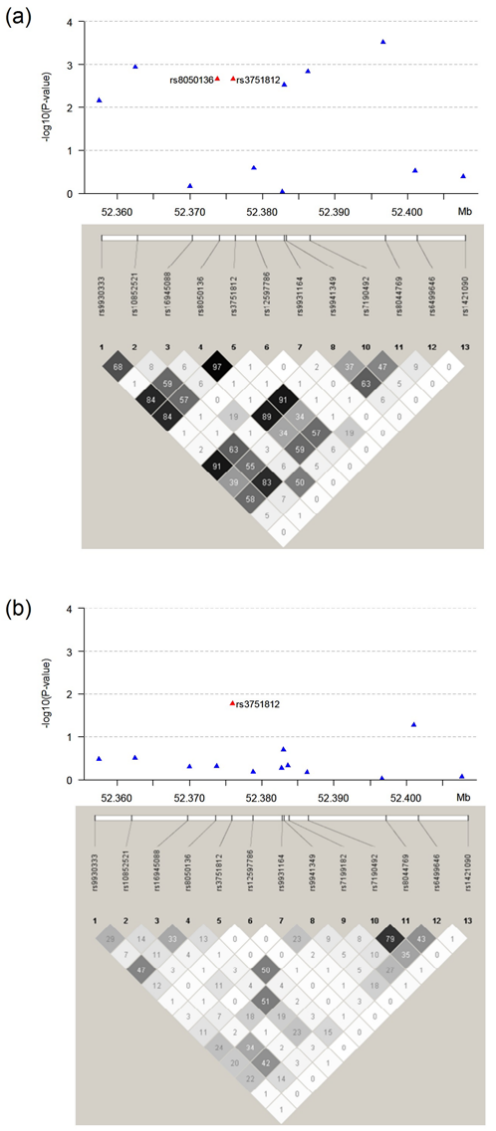
Linkage disequilibrium (r^2^) between SNPs at the *FTO* locus in (a) Caucasians and (b) African Americans: Plotted are −log10(P-value) of allelic chi-squared tests. The strength of linkage disequilibrium (r^2^) between SNPs is represented both numerically and by the depth of shading

These results demonstrate the impact of variation at this locus in AA and how we have refined the signal to rs3751812, such that it is closer to the causative underlying mutation (Figure 1). This is as a consequence of LD structural differences between African and Caucasian ancestries, as seen in our cohort (Figure S1) and in the HapMap (Figure S2).

## Discussion

From an interim analysis of our ongoing GWA study of childhood obesity, we have investigated variation in the *FTO* locus previously reported to be associated with both the adult and pediatric forms of the disease[34]. Consequently, we have replicated association of this gene with obesity by further demonstrating its effect in the childhood form of the disorder. More specifically, the common non-coding variants, rs8050136 and rs3751812 (perfect surrogates for the previously reported rs9939609 i.e. both r^2^ = 1 in Caucasians), were shown to confer risk for the disorder with comparable odds ratios to that previously observed.

Although the size of the cohort in the original genome wide association study was larger[34], the amount of testing in our cohort was restricted to a focused effort of specifically investigating if SNPs in the *FTO* gene also yield association in the same direction as previously reported. Our cohort is therefore sufficiently powered to ask this straight-forward validation question.

As the association we observe is indeed of a very similar magnitude to that of the original reports[34], [37], this independent replication confirms *FTO* as a genuine childhood obesity susceptibility gene. As such, the “winner's curse” that is often seen for other complex trait susceptibility genes[41] is not observed in this instance.

What is worth particular note is that the association observed in children is almost identical to that of adults. With the gene-environment interaction[42] models in mind, we have been motivated to look at the genetics of childhood disease in order to more readily distill the genetic component in these phenotypes due to the fact that environmental exposure and impact has been for a relatively short period of their lifetime. However, with the magnitude of the association being so comparable between children and adults, this particular research outcome suggests that the environmental interaction with this variant over time is negligible and in fact this variant may be primarily associated with early onset obesity.

Studying populations of different ancestry will also help us to globally identify and understand the genetic and environmental factors associated with estimates of obesity, as variants found in populations of both African and Caucasian ancestry may represent more universally important genes and pathways for subsequent diagnosis, prevention and treatment of obesity and its complications. As such, a cohort of African ancestry in many instances can aid in refining the anticipated association(s) made in our GWA approach. Therefore, analysis of key markers specifically in an AA cohort is part of our study design in order to help investigate if any of the association signals can be refined and localized further due to lower LD in this ethnicity. Such an instance occurred for *FTO* in our AA cohort of obese children and controls. Self-reported African ethnicity proved to be accurate, as the resulting genomic inflation factor was only 1.06. SNP rs3751812 yielded association, whereas the other SNP rs8050136, which showed evidence of association in Caucasians, was not associated in the AA cohort.

In addition, the other 11 SNPs at the locus failed to show any evidence of association in the AA cohort. These results demonstrate the impact of variation at this locus in AA and how we have refined the signal to rs3751812, which it is potentially closer to the causative underlying mutational event. Figure 1 demonstrates this point, where in Caucasians there are multiple SNPs in LD showing nominally significant association (Figure 1a) while in the AA, all but one falls away (Figure 1b). Interestingly, the associated rs3751812 is not in strong LD with the originally reported rs9939609 in African ancestry populations (r^2^ = 0.058) while rs8050136, which failed to show association in AA, is in strong LD (r^2^ = 0.819). However, the minor allele frequency of rs3751812 differs substantially between the two ethnicities, suggesting that the underlying causative variant is being tagged differently by this SNP in these populations.

Importantly, if investigators only analyze rs9939609 in their cohorts of African ancestry, they would miss the association of obesity with this locus, as has already been reported[43]. As such investigators should test both rs3751812 and rs9939609 in their obese cohorts around the world in order to thoroughly assess the influence of this locus on the phenotype globally. For example, Li *et al* [44] showed no association of rs8050136 and rs9939609 with obesity in a Chinese population so testing rs3751812 in these populations needs to be addressed before strong conclusions can be drawn on the role of variation in *FTO* in obesity predisposition in this ethnicity.

This is similar to what occurred in the case of association of variants in the *TCF7L2* gene with T2D. In Caucasians, one microsatellite and five SNPs were reported to capture the association[27] while all but one failed to show association in a T2D cohort from West Africa, namely rs7903146[45], thus also getting closer to the underlying variant.

Our results lend further support for the role of the *FTO* gene in obesity, suggesting that interventions at the FTO pathway level may be of value in patients who suffer from this disease. The *FTO* gene encodes the fat mass and obesity associated protein, *Fatso* and has been recently shown to be a 2-Oxoglutarate–Dependent Nucleic Acid Demethylase[46]. The variants that we observe association to may directly dictate splicing or some other regulatory mechanism but more likely are in LD with the causative variant(s).

Once our GWA study is complete, we will have the opportunity to look for other variants in the genome that are associated with childhood obesity, as a consequence of our use of a high resolution BeadChip. In addition, we will explore the *FTO* gene further to elucidate other potential variants that may confer genetic susceptibility to this disorder in our cohort.

## Materials and Methods

### Study Subjects

All subjects were consecutively recruited from the Greater Philadelphia area from 2006 to 2007 at the Children's Hospital of Philadelphia. Our study consisted of 418 Caucasian obese children (BMI≥95th percentile), 2,270 Caucasian controls (BMI<95th percentile), 578 AA obese children and 1,424 AA controls (BMI<95th percentile). All of these participants had their blood drawn in to an 8 ml EDTA blood collection tube and were subsequently DNA extracted for genotyping. BMI≥95th percentile was defined using the Center for Disease control (CDC) z-score = 1.645 (http://www.cdc.gov/nchs/about/major/nhanes/growthcharts/datafiles.htm). All subjects were biologically unrelated and were aged between 2 and 18 years old. All subjects were between −3 and +3 standard deviations of CDC corrected BMI i.e. outliers were excluded to avoid the consequences of potential measurement error or Mendelian causes of extreme obesity. This study was approved by the Institutional Review Board of the Children's Hospital of Philadelphia. Parental informed consent was given for each study participant for both the blood collection and subsequent genotyping.

### Genotyping

We performed high throughput genome-wide SNP genotyping using the Illumina Infinium™ II HumanHap550 BeadChip technology[47], [48] in the same manner as our center has reported previously[49]. The resources available for this project included the Illumina technology platform itself plus nine Tecan pipetting robotic systems, eight scanners, a laboratory information management system (LIMS) and automated allele-calling software. The workflow was robotic-based for automatic sample processing and included algorithms for quality control of genotypes. The facility infrastructure had sufficient computational power and servers for data processing and storing, including a series of computers that were integrated (warehouse setting) to perform continuous datamining of all gathered and generated datasets.

### Analysis

SNP rs9939609 was not included on the Illumina 550K BeadChip. However, we searched the HapMap database, and found that two SNPs, which are present on our 550K BeadChip, rs8050136 and rs3751812, are in complete LD with rs9939609 (both r^2^ = 1) in the CEU sample. Therefore, we queried the data with a test for these two perfectly correlated SNPs to investigate if they are associated with obesity in our pediatric cohort. In addition, we queried data for 11 additional SNPs that are in the same region of LD as rs8050136 and rs3751812 in the Caucasian samples. All statistical analyses were carried out using the software package *plink* (http://pngu.mgh.harvard.edu/purcell/plink/index.shtml)[50]. For the case-control study, the single marker association analysis for the genome-wide data was carried out using the 1-df allelic chi-squared test. Odds ratios and the corresponding 95% confidence intervals were calculated for each SNP. All 13 *FTO* SNPs were in Hardy-Weinberg equilibrium in both the cases and controls.

## Supporting Information

Table S1 Haplotype frequencies in the Caucasian study cohort
(0.02 MB PDF)

Table S2 Quantitative analysis of BMI results for FTO region markers
(0.04 MB PDF)

Figure S1 FTO region of LD in cohorts in the study
(0.07 MB PDF)

Figure S2 FTO region of LD in the relevant cohorts from the HapMap project
(0.06 MB PDF)
