# Supplementary material for: Association Analysis of the FTO Gene with Obesity in Children of Caucasian and African Ancestry Reveals a Common Tagging SNP
Source: PLoS One. 2008 Mar 12;3(3):e1746. doi: 10.1371/journal.pone.0001746 (PMC2262153; doi:10.1371/journal.pone.0001746)
Supplement: Table S1 — Haplotype frequencies in the Caucasian study cohort (0.02 MB PDF) [file pone.0001746.s001.pdf]

Table S1. Haplotype frequencies in the Caucasian study cohort.

Haplotype												Frequency
G	C	A	A	T	C	A	T	G	C	T	T	0.305
T	T	A	C	G	C	A	C	A	T	T	T	0.237
G	C	A	A	T	C	A	T	G	C	T	C	0.088
T	T	A	C	G	C	A	C	A	T	T	C	0.085
T	T	A	C	G	C	A	C	G	T	T	T	0.081
T	C	G	C	G	C	A	C	G	C	C	T	0.053
T	T	A	C	G	C	A	C	G	T	T	C	0.049
G	C	G	C	G	C	G	T	G	C	T	T	0.019
T	C	G	C	G	C	A	C	G	C	C	C	0.014
G	C	A	C	G	C	A	C	G	T	T	T	0.013
T	T	A	C	G	C	A	C	A	C	T	C	0.013
T	C	A	C	G	T	A	C	G	C	C	T	0.010
