# Supplementary material for: Association Analysis of the FTO Gene with Obesity in Children of Caucasian and African Ancestry Reveals a Common Tagging SNP
Source: PLoS One. 2008 Mar 12;3(3):e1746. doi: 10.1371/journal.pone.0001746 (PMC2262153; doi:10.1371/journal.pone.0001746)
Supplement: Table S2 — Quantitative analysis of BMI results for FTO region markers (0.04 MB PDF) [file pone.0001746.s002.pdf]

Table S2. Quantitative analysis of BMI results for *FTO* region markers perfectly correlated with Frayling *et al* in Caucasians (**bold**) plus all other markers present on the BeadChip in the corresponding HapMap CEU LD block in our (a) Caucasian and (b) African American cohorts respectively.

(a) QTL for BMI in the Caucasian cohort

Chr	SNP	B35 location	Minor Allele	Major Allele	NMISS	BETA	SE	R2	T	P
16	rs9930333	52357478	T	G	2630	0.08648	0.03191	0.002787	2.7100	0.0068
16	rs10852521	52362466	T	C	2632	-0.06088	0.0316	0.001409	-1.9260	0.0542
16	rs16945088	52370025	G	A	2630	-0.07592	0.05542	0.0007135	-1.3700	0.1709
**16**	**rs8050136**	**52373776**	**A**	**C**	**2625**	**0.1037**	**0.0323**	**0.003919**	**3.2120**	**0.0013**
**16**	**rs3751812**	**52375961**	**T**	**G**	**2610**	**0.09931**	**0.03231**	**0.00361**	**3.0740**	**0.0021**
16	rs12597786	52378808	T	C	2631	0.02254	0.1121	1.54E-05	0.2010	0.8407
16	rs9931164	52382739	G	A	2633	-0.003899	0.118	4.15E-07	-0.0331	0.9736
16	rs9941349	52382989	T	C	2633	0.09884	0.03215	0.003579	3.0740	0.0021
16	rs7199182	52383621	G	A	-	-	-	-	-	-
16	rs7190492	52386253	A	G	2578	-0.06477	0.03335	0.001462	-1.9420	0.0523
16	rs8044769	52396636	T	C	2632	-0.08179	0.03163	0.002536	-2.5860	0.0098
16	rs6499646	52401034	C	T	2628	-0.02667	0.05496	8.97E-05	-0.4853	0.6275
16	rs1421090	52407671	C	T	2628	-0.03454	0.03629	0.0003448	-0.9518	0.3413

(b) QTL for BMI in the African American cohort

Chr	SNP	B35 location	Minor Allele	Major Allele	NMISS	BETA	SE	R2	T	P
16	rs9930333	52357478	G	T	1966	0.04355	3.90E-02	0.0006339	1.1160	0.2645
16	rs10852521	52362466	T	C	1968	-0.05961	0.04377	0.0009425	-1.3620	0.1734
16	rs16945088	52370025	G	A	1963	0.0123	0.04219	4.33E-05	0.2915	0.7707
**16**	**rs8050136**	**52373776**	**A**	**C**	**1960**	**0.05674**	**3.98E-02**	**0.001039**	**1.4270**	**0.1537**
**16**	**rs3751812**	**52375961**	**T**	**G**	**1962**	**0.04218**	**6.56E-02**	**0.000211**	**0.6431**	**0.5202**
16	rs12597786	52378808	T	C	1965	-0.05468	0.1285	9.22E-05	-0.4255	0.6705
16	rs9931164	52382739	G	A	1968	-0.04239	0.09297	0.0001057	-0.4559	0.6485
16	rs9941349	52382989	T	C	1967	0.01067	0.05158	2.18E-05	0.2069	0.8361
16	rs7199182	52383621	G	A	1952	0.05148	0.04276	0.0007427	1.2040	0.2288
16	rs7190492	52386253	A	G	1932	-0.03174	0.04955	0.0002126	-0.6406	0.5219
16	rs8044769	52396636	T	C	1968	-0.03779	0.04692	0.0003299	-0.8055	0.4206
16	rs6499646	52401034	T	C	1965	0.0005684	0.04068	9.95E-08	0.0140	0.9889
16	rs1421090	52407671	C	T	1968	0.02868	0.04375	0.0002185	0.6555	0.5122

NMISS: number of individuals tested; BETA: regression coefficient for the test SNP; SE: standard error of the regression coefficient; R2: r^2 value in linear regression; T: test statistic; P: *P*-value
