# Supplementary material for: Association Analysis of the FTO Gene with Obesity in Children of Caucasian and African Ancestry Reveals a Common Tagging SNP
Source: PLoS One. 2008 Mar 12;3(3):e1746. doi: 10.1371/journal.pone.0001746 (PMC2262153; doi:10.1371/journal.pone.0001746)

Figure S1. *FTO* region of LD in cohorts in the study: |D'| and r^2

FTO: Caucasians

|D'|

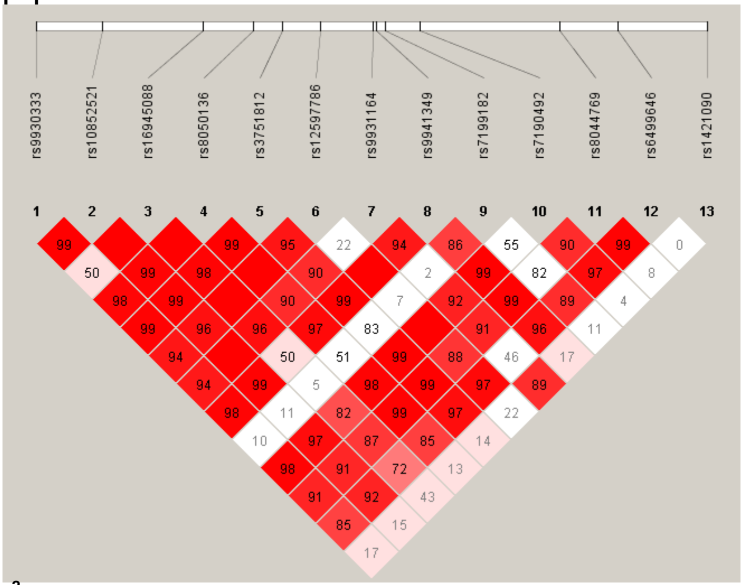

FTO: African Americans

|D'|

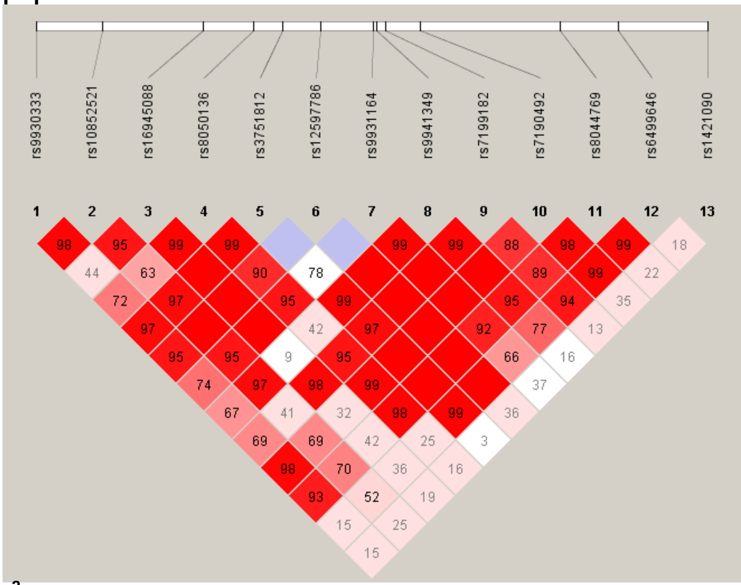

r^2

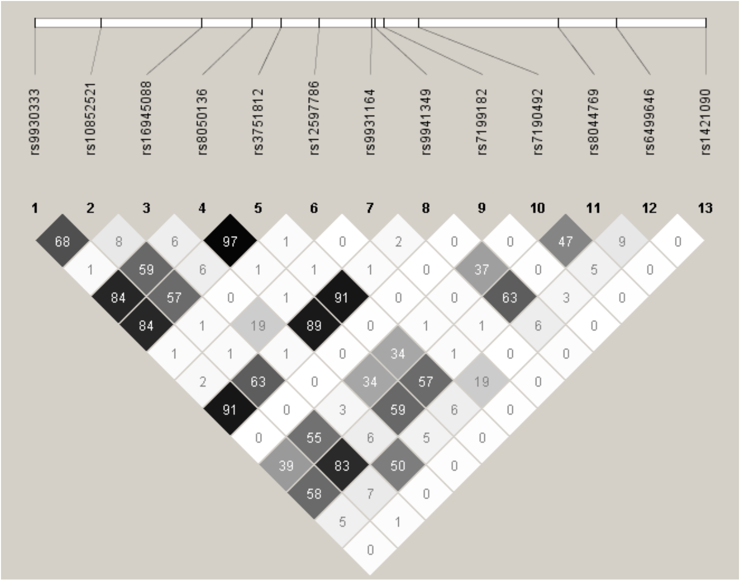

r^2

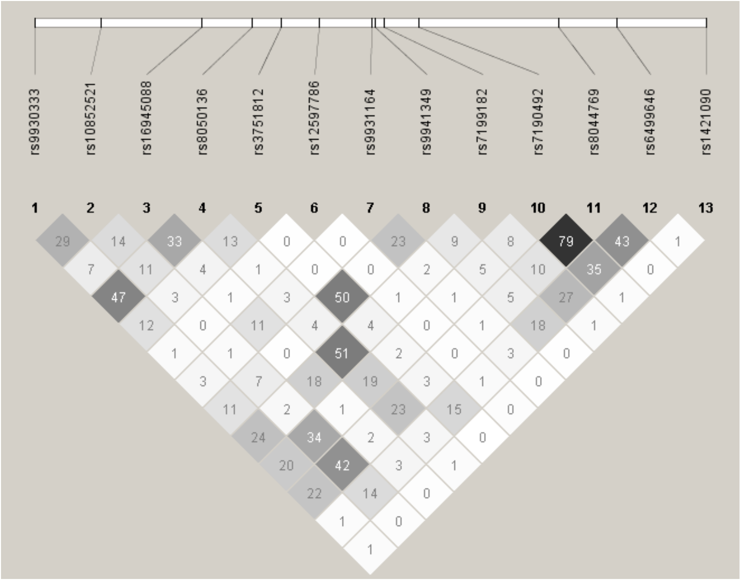

Supplement: Figure S1 — FTO region of LD in cohorts in the study (0.07 MB PDF) [file pone.0001746.s003.pdf]
