# Supplementary material for: Association Analysis of the FTO Gene with Obesity in Children of Caucasian and African Ancestry Reveals a Common Tagging SNP
Source: PLoS One. 2008 Mar 12;3(3):e1746. doi: 10.1371/journal.pone.0001746 (PMC2262153; doi:10.1371/journal.pone.0001746)

Figure S2. *FTO* region of LD in the relevant cohorts from the HapMap project: |D'| and r^2

***FTO*: CEU**
|D'|

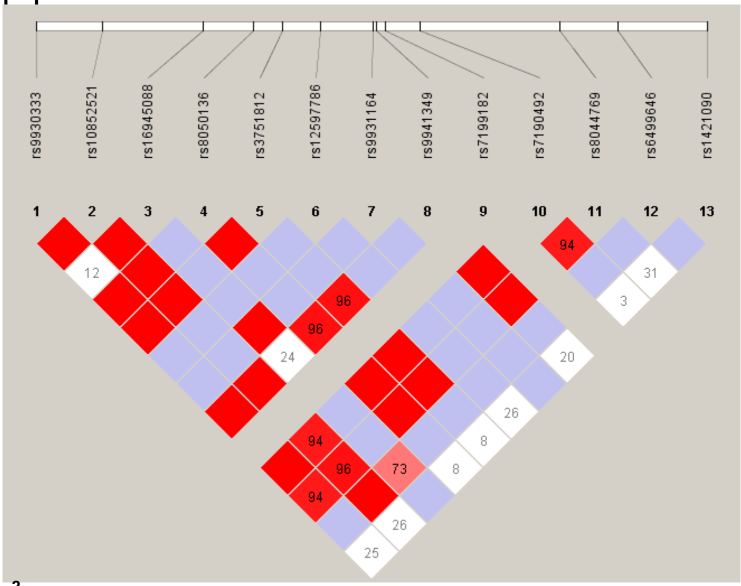

r^2

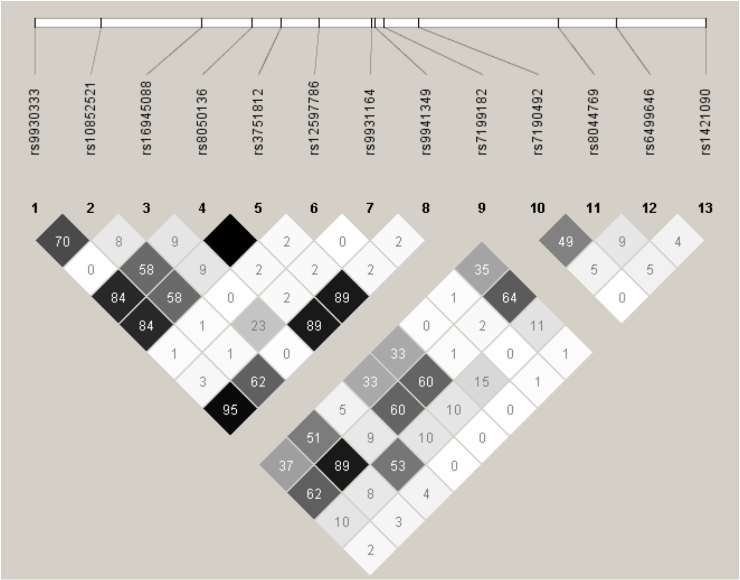

***FTO*: YRI**
|D'|

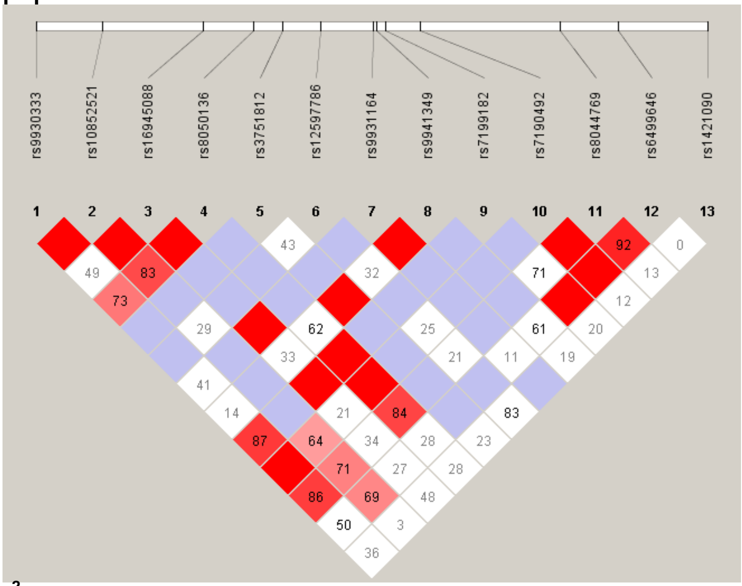

r^2

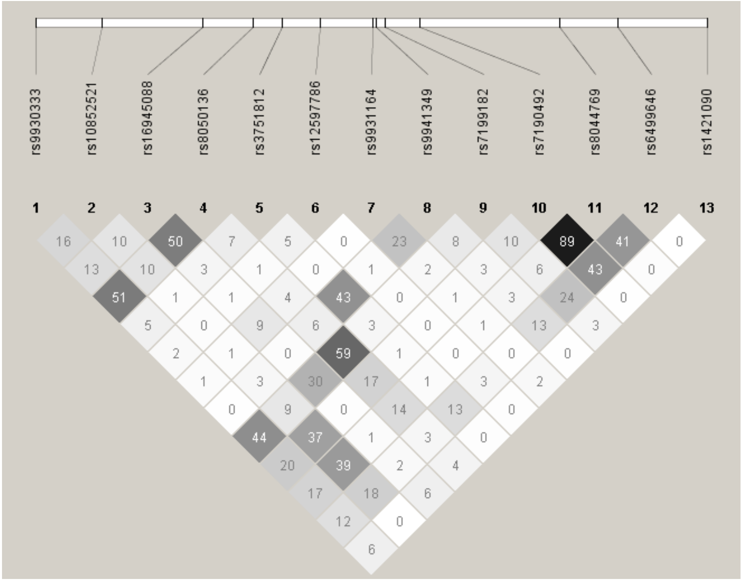

Supplement: Figure S2 — FTO region of LD in the relevant cohorts from the HapMap project (0.06 MB PDF) [file pone.0001746.s004.pdf]
